# Self-Perceived Dentists' Knowledge of Temporomandibular Disorders in Krakow: A Pilot Study

**DOI:** 10.1155/2020/9531806

**Published:** 2020-05-27

**Authors:** Magdalena Osiewicz, Paulina Kojat, Maria Gut, Zuzanna Kazibudzka, Jolanta Pytko-Polończyk

**Affiliations:** ^1^Department of Integrated Dentistry, Dental Institute, Faculty of Medicine, Jagiellonian University Medical College, Krakow, Poland; ^2^Students Scientific Group of Integrated Dentistry at the Department of Integrated Dentistry, Dental Institute, Jagiellonian University, Medical College, Krakow, Poland

## Abstract

**Introduction:**

The most common nondental orofacial pain conditions are temporomandibular disorders (TMDs). TMD basic examination and clinical management are included in a curriculum of each dentistry programme taught in Poland, but it is not clear how the dentists cope with diagnosis and possible treatment in their routine dental practices. The objective of the present study was to assess a level of self-perceived knowledge of TMD amongst dentists in Poland. *Materials and methods*. The participants, of whom all studied and graduated from a Polish university, were randomly selected from dental offices in Krakow (Poland). The selected dentists were administered an anonymous questionnaire, which contained questions measuring self-assessment of knowledge of TMD diagnosis and therapy and assessing knowledge of ethology and TMD symptoms.

**Results:**

Only 6.5% of the participants identified their TMD knowledge as very good, 32.3% assessed it as good, 39.3% thought it was sufficient, 20.4% as insufficient, and 1.49% considered it as poor. 9.4% of all participants have attempted to diagnose and treat TMD patients very often, 26.4% declared performing it often, 45.8% rarely, and 18.4% had never made such an attempt. There was a significant relationship between the dentists' knowledge and their attempts at diagnosing and treating TMD patients (*p* < 0.05).

**Conclusion:**

The level of TMD knowledge amongst the Polish dentists is still insufficient. Raising its level would considerably help the dentists to refer their patients to right specialists for a diagnosis and TMD treatment and/or interdisciplinary management of TMD patients.

## 1. Introduction

Temporomandibular disorders (TMDs) are characterized by pain of masticatory muscles (when in function), pain in the area of preauricular and/or temporomandibular joint (TMJ), limited and/or deviated mandibular movements, and TMJ sounds (i.e., clicking and/or crepitus) during function [1].

The most common nondental orofacial pain conditions are TMDs [2]. The studies of prevalence of TMD among the healthy Poles are based on two research studies: one carried out on 260 18-year-old adolescents, of whom 26.5% received one or more of TMD diagnoses according to Research Diagnostic Criteria for Temporomandibular Disorders (RDC/TMD), and second on adults [3–5]. Another research showed that the frequency of TMD diagnoses among the Polish patients was similar to that of other populations [6]. In both research studies carried out on the Polish population, myofascial pain was the most frequent diagnosis. The number of patients requiring a TMD treatment varies considerably, ranging from 1.5% to 30% [7]. A multidisciplinary approach in TMD cases' treatment is crucial and may involve dentists, physical therapists, speech pathologists, physicians, and psychologists [8].

Although, both TMD's basic examination and clinical management are in a curriculum of each dentistry programme taught in Poland, it is not clear how dentists cope with diagnosis and possible treatment in routine dental practice [9].

Unfortunately, due to the lack of previous studies, there are no data on the self-perceived knowledge level of TMD among the Polish dentists to be compared with those retrieved from other countries. Considering the scarcity of research on this topic, the objective of present study was to assess the level of dentists' self-perceived knowledge of TMD in Poland.

## 2. Materials and Methods

### 2.1. Participants

400 dental offices in Kraków (Poland) were randomly selected from Register of Entities Performing Medical Activities by the study's coordinator to randomly identify the study participants, of whom all studied and graduated from a Polish university. The selected dentists were contacted in person by one of the three dentistry students involved in the study. All participants were informed that no identifiable information will be published or released and that participation is voluntary. Each participant was given an anonymous questionnaire to be filled in a spare room, taking approximately 5–10 minutes to be completed. All data were confidentially analyzed.

Prior to the study, the participants were informed of its aim and asked to sign consent forms. The research program was approved by the Jagiellonian University Bioethics Committee (approval no. 1072.6120.83.2018KBET). The study was conducted in accordance with the recommendations of the Declaration of Helsinki. The research commenced in April 2018 and ended in August 2019.

### 2.2. Questionnaire

**Figure 1:**
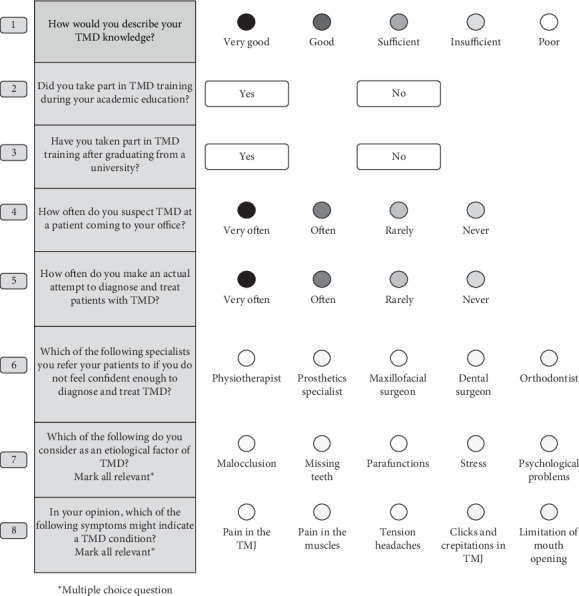
Anonymous questionnaire.

The participants were given an anonymous questionnaire containing 8 questions in total regarding: 3 questions on their self-assessment knowledge of TMD diagnosis and therapy and education in the field of TMD, 3 questions on TMD patient population and referrals, and 2 in regards to the participant's knowledge of TMD ethology and the symptoms which, in the participant's opinion, might indicate a TMD condition (Figure 1).

### 2.3. Statistical Analysis

For qualitative variables, percentages and raw counts were reported. Comparisons of qualitative variables in groups were conducted with the chi-squared test (with Yates' correction for 2 × 2 tables) or with Fisher's exact test (when low expected values had occurred). Analyses were conducted at 0.05 level of significance. *R* software, version 3.6.1, was used [10].

## 3. Results

A total of 201 volunteers participated in the anonymous study. The response rate was 50.3% (201/400).

### 3.1. Knowledge Self-Assessment and the Dentists' Education in the Field of TMD

Only 6.5% of the participants assessed their TMD knowledge as very good, 32.3% assessed it as good, 39.3% thought it was sufficient, 20.4% marked it as insufficient, and 1.5% considered it poor. 64.2% of the participating volunteers had received some training in diagnosing and/or treating TMD patients during their academic education. 50.2% of all the participants had attended some postgraduate training sessions after the graduation from a university.

### 3.2. TMD Patient Population and Referral

Being asked if ever suspected any patients of having TMD symptoms, only 9% of the dentists chose the first option very often. 55.7% selected the second option often, 31.8% chose rarely, and 2.5% chose never. 9.4% of all participants have attempted to diagnose and treat TMD patients very often, 26.4% declared performing it often, 45.8% rarely, and 18.4% had never made such an attempt. Majority of the dentists reluctant to undertake diagnosis and implement some treatment for patients being suspected of TMD refers these patients to prosthetics specialists (56.7%). Some dentists refer their patients to physiotherapists (32.8%), and some others to maxillofacial surgeons (2%), dental surgeons (2.5%), and hardly never to orthodontists (1.5%) (Table 1).

### 3.3. Ethology and the Symptoms of TMD

When asked of major causes of TMD, almost all participants (93.5%) indicated stress as the main one. Similarly, 92% of participants thought that missing teeth were to blame, 90% chose parafunction as the main cause of TMD, 86.6% selected malocclusion, whereas 75.6% of dentists blamed psychological factors. If it comes to the syndromes, TMJ pain was selected as the most frequent (96.5%), followed by sounds in the TMJ area (92.5%), myofascial pain (90.5%), and tension headache (86.6%). A limitation of mouth opening (87%) was the least often symptom chosen. Next, the relationship between the questions was examined. There was only one significant relationship (as *p* < 0.05): the better the dentists' knowledge, the more often they attempt to diagnose and treat TMD patients (Table 2).

## 4. Discussion

The aim of the study was to assess the level of the self-perceived knowledge of diagnosing and treating TMD among the Polish dentists and to assess their knowledge of TMD ethology and symptoms.

According to the Regulation of the Minister of Health 2017 [11] regarding standard graduate academic program of practical classes for doctors and dentists, the practical classes in prosthodontics include patients' examination and diagnosis, prevention and treatment deriving from tooth loss, malocclusion, TMDs, and other disorders in uncomplicated clinical cases. Yet, the present study shows that only 6.5% of the participants described their knowledge of TMD as very good, whereas almost a quarter considered it as insufficient or poor. The result is similar to results of a research carried out in Germany, which tested dentists' confidence in diagnosing orofacial pain. As few as 2% of German dentists thought it was very good, whereas 35% considered it insufficient or poor [12].

About half of the Polish respondents (50.2%) agreed to participate in the postgraduate training in diagnosing or treating TMD, which corresponds with a study carried out in Sweden (51%) [13]. However, in Germany, 41% dentists took part in postgraduate courses [12].

The study clearly demonstrated that there was a significant relationship (*p* < 0.05) between the level of knowledge and attempts to diagnose and treat TMD: the better the knowledge, the attempts have been made more often. Unfortunately, due to the dentist's insufficient knowledge, patients with TMD are often misdiagnosed, which means they have to undergo various treatments for nonrelated disorders, and are referred to other specialists without a clear idea of who they should be referred to, which often leads to frustration, lack of satisfaction, and a compromised quality of life [14].

The Polish dentists who do not attempt to diagnose and treat patients suspected of TMD often refer them to a specialist in prosthetics instead. The latter were given over several months long training in TMD patients' diagnosis and treatments comparing to other medical specializations. However, the fact that over one-third of the examined dentists refers TMD patients for diagnosis and treatment to physiotherapists is worrying. Orofacial pain has a prevalence of about 10% in the general population, and many conditions share similar clinical features [15]. TMD occurrence has been reported in patients with some chronic pain conditions or psychological disorders and also life-threatening diseases, such as Lyme disease. Hence, the diagnoses should be carried out by a dentist who might be the first one to diagnose a patient's disease and refer the patient to a right specialist being able to prescribe without any further delay the right treatment [16–21]. It is recommended to implement an interdisciplinary management of TMD patients, involving dentists, physical therapists, psychologists, ear/nose/throat specialists, and speech pathologists, especially when the pain is chronic [8].

The majority of the Polish dentists consider stress, parafunction, and psychological factors to be the main causes of TMD. 96% of the American dentists are convinced that stress plays an important role in causing TMD, 100% of Korean dentists agree with them, 88% of Mexican dentists, and 88% of Swedish agree too [22–25].

On the other hand, a similar number of the Polish dentists disagree with the idea, claiming that missing teeth and malocclusion are to be blamed. The results of this study are similar to those of Lopez-Frias et al.'s study carried out among the Spanish dentists, where 98.5% of respondents believed that occlusal alterations are accountable for TMD [26]. In 1934, the dental profession was drawn into the area of TMD because of the article written by James Costen, an otolaryngologist [27]. On the basis of eleven cases, he suggested that changes in a dental condition (e.g., over closure of bites, lack of molar support, and malocclusion) were responsible for various symptoms, such as impaired hearing, stuffy ears, tinnitus, masticatory muscle and joint pain, dizziness, sinus symptoms, and headache. Because of that for years, the focus of dental professionals' approach to patients with TMDs has been solely based on the assessment and correction of purported abnormalities of the occlusion [28]. Over the last few years, there has been a visible accumulation of evidence against using irreversible mechanical therapies in TMD treatment, in favour of bio-psycho-sociological approach to TMD. Besides physiological overloading, many epidemiological studies have demonstrated the existence of a strong relationship between TMDs and psychopathology, and the occlusal factors should no longer be taken into consideration in TMD diagnosis and treatment [29, 30].

According to a Polish graduate curriculum, the topic of TMD is included in prosthetics studies, which covers 285 hours of lectures and clinical classes [11, 31]. Yet, only one lecture and one practical class are dedicated to TMD management. According to the record book of clinical student training, after the class a student should “know the rules of performing and should be able to provide assistance to the initial treatment of TMD.” This is the reason why the dentists are reluctant to diagnose and treat patients with TMD. It is not a surprise that the graduates do not feel prepared well enough to carry out a treatment they have never done before. In contrary, Swedish students have separate classes dedicated only to TMD, which prepare them to treat TMD patients far better than those in countries where very little time is given to TMD [32].

As the final remark, it must be pointed out that this is the first study on the self-perceived dentists' knowledge of TMD in Central/East Europe. Generalization of findings may be limited by the sample of dentists included in this study, which might have influenced the representativeness of the sample, with respect to the general Polish dentist population. The crucial limitation of the study is the lack of information about the demographics of the participants such as gender, age, lack of specialty, and years of experience. It is suggested to separate specialists and general dentists in future studies as well as take into consideration their work experience. In terms of limitations, the study was carried out on a relatively small group of dentists; therefore, it should be considered as a pilot study. In further studies, the sample group should be expanded to volunteers from other cities allowing for a cross-cultural comparison. Therefore, the presented results should be interpreted with caution and some further studies based on a bigger sample and including different dental specialties are recommended.

## 5. Conclusions

The study is the first one in Central Europe carried out among the Polish dentists with the use of a questionnaire. The results are within the range of those from other countries. However, Polish dentists' knowledge of TMD is still insufficient. Increasing TMD knowledge level among the dentists would considerably help them in referring their patients to the right specialist for further diagnosis and TMD treatment and/or interdisciplinary management of TMD patients. Therefore, it is very important to design a suitable study programme which would provide graduate dentists with necessary practice and knowledge of TMD.

## Figures and Tables

**Table 1:** Descriptive statistics of questionnaire.

Question	Answer	*n* (%)
(1) How would you describe your TMD knowledge?	Very good knowledge	13 (6.5%)
	Good knowledge	65 (32.3%)
	Sufficient knowledge	79 (39.3%)
	Insufficient knowledge	41 (20.4%)
	Poor knowledge	3 (1.5%)

(2) Did you take part in TMD training during your academic education?	Training during study	129 (64.2%)
	No training	71 (35.3%)
	No answer	1 (0.5%)

(3) Have you taken part in TMD training after graduating from a university?	Postgraduate training	101 (50.2%)
	No training	98 (48.8%)
	No answer	2 (1%)

(4) How often do you suspect TMD at a patients coming to your office?	Very often	18 (9%)
	Often	112 (55.7%)
	Rarely	64 (31.8%)
	Never	5 (2.5%)
	No data	2 (1%)

(5) How often do you make an actual attempt to diagnose and treat patients with TMD?	Very often	19 (9.4%)
	Often	53 (26.4%)
	Rarely	92 (45.8%)
	Never	37 (18.4%)

(6) Which of the following specialists you refer your patients to if you do not feel confident enough to diagnose and treat TMD?	Physiotherapist	66 (32.8%)
	Prosthetics specialist	114 (56.7%)
	Maxillofacial surgeon	4 (2%)
	Dental surgeon	5 (2.5%)
	Orthodontist	3 (1.5%)
	No answer	9 (4.5%)

(7) Which of the following do you consider as an etiological factor of TMD? Mark all relevant.^*∗*^	Malocclusion	174 (86.6%)
	Missing teeth	185 (92%)
	Parafunctions	181 (90%)
	Stress	188 (93.5%)
	Psychological problems	152 (75.6%)

(8) In your opinion which of the following symptoms might indicate a TMD condition? Mark all relevant.^*∗*^	Pain in the TMJ	194 (96.5%)
	Pain in the muscles	182 (90.5%)
	Tension headaches	174 (86.6%)
	Clicks or crepitations in TMJ	186 (92.5%)
	Limitation of mouth opening	175 (87%)

^*∗*^Multiple choice question.

**Table 2:** The relationship between the dentists' knowledge and their attempts at diagnosing and treating TMD patients.

Attempts to diagnose and treat	Knowledge				*p*
	Very good (*N* = 13)	Good (*N* = 65)	Sufficient (*N* = 79)	Insufficient or poor (*N* = 44)	
Very often	7 (53.85%)	6 (9.23%)	6 (7.59%)	0 (0.00%)	*p<*0.001^*∗*^
Often	5 (38.46%)	25 (38.46%)	14 (17.72%)	9 (20.45%)	
Rarely	0 (0.00%)	27 (41.54%)	40 (50.63%)	25 (56.82%)	
Never	1 (7.69%)	7 (10.77%)	19 (24.05%)	10 (22.73%)	

^*∗*^ *p* value was calculated by using Fisher's exact test (in case of low expected values).

## Data Availability

The data used to support the findings of this study are available from the corresponding author upon request.
